# A Simple Introduction to the Clinical Chemistry of the Urine

**Published:** 1846-07

**Authors:** 


### *A Simple Introduction to the Clinical Chemistry of the Urine.*

The chemistry of the urine, as modified by disease, is becoming a very important element in pathology and diagnosis. Many practitioners are repelled from minute attention to this field of symptoms by the impression that it requires minute acquaintance with analytical chemistry, and a practical familiarity with chemical manipulations. We suspect, however, that enough knowledge of the subject, at least for all important practical purposes, may be acquired with but little effort, and, therefore, the prevailing inattention is less excusable than is generally supposed. This appears to have been the view taken by the writer of the following article, which we take from the British American Journal of Medical and Physical Science.—*Ed. Buff. Journal.*

Under this title, a communication from an anonymous correspondent, appears in a late number of the *Medical Gazette.* The directions which it gives for this investigation are sufficient for all practical purposes. Its perusal, in an abbreviated form, will repay the reader.

"The urine should, for all purposes of examinatiou, be collected clear from admixture of dust or other impurities. The morning's urine is generally employed, as it is difficult to obtain that passed during the whole twenty-four hours, but the quantity passed during this space of time should be noted; and, also, it should be ascertained whether this is greater or less than the quantity usually passed by the patient during that space of time. The note should also state what urine was examined, whether the morning's, or that passed in the course of the day.

"The characters of the fluid should then be observed — best in the glass in which the specific gravity is presently to be taken; its reaction on test-paper, and any sediment that it may have deposited. The latter should be examined under the microscope. The acid and alkaline reaction is shown by blue and red litmus-paper.

"If the urine is acid, turbid, with a red deposite staining the sides of the vessel, we know at once that the urates are in excess; if alkaline, or even acid, of a pale colour, slightly turbid, with a light cloud floating in it, and an iridescent pellicle on the top, we presume the phosphates to be in excess, and this the more if the urine is highly offensive to the smell, and full of mucous strings. A clear pale urine may be found in connexion with hysteria, with Bright's disease of the kidney, or with diabetes. A smoky colour is very indicative of Bright's disease; (a yellowish tint on the sides of the vessel, or communicated to linen; indicates the presence of bile ;) and a greenish tint should remind us to look for crystals of oxalate of lime.

"The specific gravity is best estimated by means of a common hydrometer, made for the purpose, contained in a strong glass tube, in which it may be floated when required. It should be allowed to sink gently down to its level, as all the fluid that collects upon it higher up tends to weigh it down, and makes the urine seem of lighter specific gravity than it really is. High specific gravity, that is to say, all above 1024, may denote diabetes, or may result from the patient, at the time, employing diuretic salts. Low specific gravity, or below 1014, may be connected with granular degeneration of the kidney. The specific gravitv, in connexion with what has been already noted, concerning the color and reaction of the fluid, is to guide us in the subsequent application of our tests.

"Heat and nitric acid are, for most purposes, enough ; their effects are best witnessed in common test tubes, into which the urine may readily be poured, even from a large vessel, by using one tube as the guide, down the side of which the fluid may run into the other.

"Urine of a low specific gravity had better be heated, and if a precipitate forms, a few drops of nitric acid should be added. If the precipitate re-dissolves, it is to be considered indicative of the presence of the phosphates in excess, if it do not dissolve, but be rather increased by the addition of the acid, it is albumen. It is to be remembered that a precipitation of the phosphates by heat may take place as well in acid as in alkaline urine.

"To urine of a higher specific gravity, nitric acid may be added at once. Every precipitate that forms may be uric acid or albumen. To determine this, the fluid should be heated, when the precipitated uric acid will be re-dissolved, the albumen will remain coagulated. A partial or entire solution of the precipitate, by long-continued heat, may denote so interesting a form of disease, that that the beginnner would do well to call in the aid of a more experienced chemist under such circumstances. The results thus obtained should not be lost; the tubes must be set by for a day, and then it should be noted, by the aid of a common pocket measure, how high the precipitate stands in the fluid, occupying, for example, half, a third, or an eighth part, as the case may be. The quantity of little crystals of uric acid that have dusted over the inside of the tube should also, at the same time, be looked to.

"If precipitate forms, a reference to the specific gravity must tell us whether there is any thing more to be expected, or whether we may presume that we are dealing with healthy urine. If the specific gravity be high, and the re-agents have produced only slight effects, the nature of the case may tell us whether it be worth while to look for sugar in the urine. This is most satisfactorily effected by means of fermentation; about two drachms of the urine being introduced, with a little yeast, into a phial with a perforated cork, through which the longer leg of a bent tube passes, and the whole inverted in a cup of water, where another phial, filled with water and inverted, is to receive the short leg of the tube. At a temperature above 60°, any sugar that may be present will begin to be decomposed, and carbonic acid will collect and remain for some time unabsorbed in the other phial. If no gas collects, there is no sugar in the urine; but there may have been albumen which we have failed to detect from not adding enough acid, for a little nitric acid only renders the albumen uncoagulable; forms, in fact, a soluble nitrate, which is not precipitated by heat. A few additional drops of nitric acid will prevent any error from this cause, by at once precipitating the albumen.

"It remains now to consider those cases where the urine evidently contains some soluble matter in excess, which, however, is neither albumen nor uric acid, and to notice a few appearances which are sometimes perplexing to a beginner. A free effervescence may arise either from the presence of the carbonate of an alkali, or the decomposition of urea, or that of uric acid. The first case might be solved by looking to see what the patient is taking, all neutral salts, with vegetable acids, being converted into carbonates of that base during their passage into the urine. The second is best determined by setting aside two drachms of urine in a saucer, with about a quarter of its volume of nitric acid, of course, without having applied heat, when the appearance of crystals denotes an excess of urea. The third is best solved by adding a few drops of any acid to the urine in a tube, and observing, as before recommended, the number of crystals that have formed by the ensuing day.

"The flaky precipitates, recognized to be albumen, may be seen sometimes yellow, sometimes red or pink, or covered with air bubbles. For the present, it is enough to state, that these appearances result from the decomposition of the uric acid, by the nitric acid, which deepens the color of the urine, or of the urea, or any substances, as above, which effervesce on the addition of this reagent; or; lastly, the yellow colour may arise from their being nothing present to prevent the nitric acid producing this its ordinary effect upon animal substances.

"The sum of these remarks is this. That the urine deviates from the condition of health most commonly in a few particular ways, whether by an excess or deficiency of its normal constituents, or by the presence of matters which should not exist at all in the urine."

The chemical study of the morbid conditions of the urine is thus shown not to be so difficult as many have believed it to be. Without attention to them, our therapeutics are a dead letter, and here are the means of preventing such a result: they are sufficiently simple and ready of application.—*Lancet.*

